# Formation and Band Gap Tuning Mechanism of Multicolor Emissive Carbon Dots from *m*‐Hydroxybenzaldehyde

**DOI:** 10.1002/advs.202300543

**Published:** 2023-04-21

**Authors:** Yan Li, Can Liu, Hao Sun, Menglin Chen, Defa Hou, Yunwu Zheng, Haijiao Xie, Bei Zhou, Xu Lin

**Affiliations:** ^1^ National Joint Engineering Research Center for Highly‐Efficient Utilization Technology of Forestry Resources School of Materials and Chemical Engineering, Southwest Forestry University 300 Bailong Road Kunming Yunnan 650224 P. R. China; ^2^ Hangzhou Yanqu Information Technology Co., Ltd. Y2, 2nd Floor, Building 2, Xixi Legu Creative Pioneering Park, No. 712 Wen'er West Road, Xihu District Hangzhou Zhejiang Province 310003 P. R. China

**Keywords:** aggregation, carbon dots, fluorescent mechanism, *m*‐hydroxybenzaldehyde, multicolor emission, supramolecular

## Abstract

Reported in 2004, carbon dots (CDs) have been widely used in various fields due to their excellent optical properties. However, the mechanism of their fluorescence modulation is still a controversial issue, which also seriously affects the further development of carbon dots. In this paper, *m*‐hydroxybenzaldehyde is used as a raw material to obtain multicolor luminescent CDs by pyrolysis under different reaction conditions, thereby revealing the forbidden band tuning and formation mechanism of CDs. Different acid–base conditions lead to different reaction paths of the precursors, forming molecular fluorophores with different conjugated structures, which aggregate to eventually form CDs and further enhance the photoluminescence of the system by inhibiting the movement of the fluorescent centers.

## Graphical abstract

Herein, the mechanisms of band gap tuning and formation of CDs based on the pyrolysis of *m*‐hydroxybenzaldehyde are revealed. Different acid–base conditions lead to different reaction paths from the precursor and the formation of fluorophores with different conjugated structures. These fluorophores aggregate to form CDs, which further enhance the photoluminescence of the system.

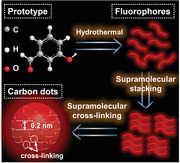

## 1 Introduction

Carbon dots (CDs) are quasi‐spherical nanoparticles smaller than 10 nm, and their internal structure is generally composed of sp^2^‐ and sp^3^‐hybridized carbon atoms.^[^ ^1^, ^2^, ^3^ ^]^ Because of their unique fluorescence characteristics, CDs are widely used in photovoltaic energy devices, information encryption and anticounterfeiting, biological imaging and medical treatment, sensor detection, and other fields.^[^ ^4^, ^5^, ^6^, ^7^ ^]^ However, the mechanism of their fluorescence regulation is still a controversial issue, seriously affecting the further development of CDs. At present, the quantum size effect,^[^ ^8^, ^9^ ^]^ surface defect state,^[^ ^10^ ^]^ molecular state,^[^ ^11^ ^]^ and other fluorescence mechanisms^[^ ^12^ ^]^ have been proposed, but the relationship between the CDs structure and fluorescence characteristics remains a mystery, which has become a focus of basic research on CDs.^[^ ^13^, ^14^, ^15^ ^]^

The biggest difficulty in analyzing the mechanism of regulation of the fluorescence color is that the CDs structure cannot be determined.^[^ ^16^, ^17^ ^]^ In general, CDs are synthesized from more than two precursors at high temperature, resulting in a complex product structure.^[^ ^18^, ^19^ ^]^ Moreover, precursors such as phenylenediamine do not undergo a clear reaction between functional groups,^[^ ^20^ ^]^ making it difficult to characterize the structure by nuclear magnetic resonance (NMR) analysis, leading to disputations regarding the exact structure and luminescence mechanism of CDs.^[^ ^21^, ^22^, ^23^, ^24^ ^]^ Therefore, the regulatory mechanism can be described equivocally as follows: different sp^2^ domain structures lead to different colors of fluorescence in CDs nuclei.^[^ ^25^, ^26^ ^]^ How are CDs structures formed?^[^ ^27^, ^28^ ^]^ How does the CDs structure affect the fluorescence color and quantum yield?^[^ ^29^, ^30^ ^]^ These questions urgently need to be addressed to re‐evaluate the formation mechanism of CDs and their related reactions in the regulation of fluorescence color.^[^ ^31^, ^32^ ^]^

**Figure 1:**
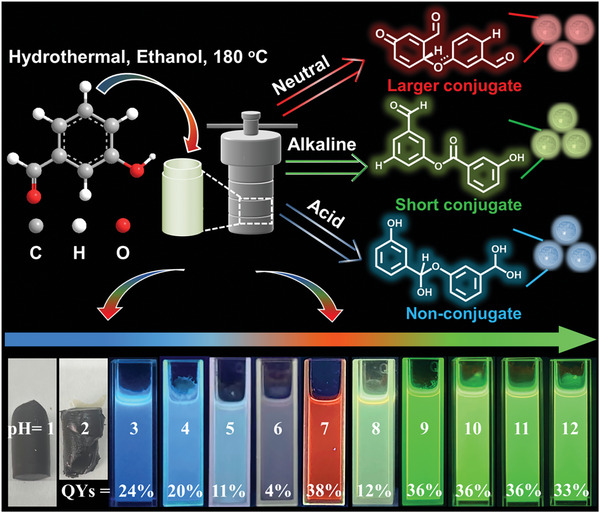
Preparation of RGB‐CDs from *m*‐hydroxybenzaldehyde under different reaction conditions.

Herein, we reveal the band gap tuning and formation mechanisms of CDs based on a prototype involving pyrolysis of *m*‐hydroxybenzaldehyde. *m*‐Hydroxybenzaldehyde contains aldehyde and hydroxyl groups; a clear chemical reaction can occur between functional groups, and simply changing the conditions in the pyrolysis process can result in the synthesis of multicolor emissive CDs, which are useful for the structural analysis of CDs (**Figure** **1**). Therefore, red, green, and blue emissive CDs were obtained from *m*‐hydroxybenzaldehyde under neutral, alkaline, and acidic conditions, respectively (Figure S1, Supporting Information). We also tried other isomers of hydroxybenzaldehyde, but the *o‐* and *p*‐isomers could not form RGB‐CDs when the reaction conditions were changed (Figure S2, Supporting Information). The transmission electron microscope (TEM) images revealed that the CDs were well dispersed, and the average particle size of the three differently colored CDs was 3.5 nm (Figure S3, Supporting Information), indicating that the quantum size effect was not the dominant mechanism responsible for the chromatic emissions. High‐resolution TEM images of the samples showed well‐resolved lattice fringes for graphite carbon with an interplanar spacing of 0.20 nm, which indicated the existence of a supramolecular‐stacked structure in the CDs.^[^ ^33^, ^34^ ^]^

## 2 Results and Discussion

**Figure 2:**
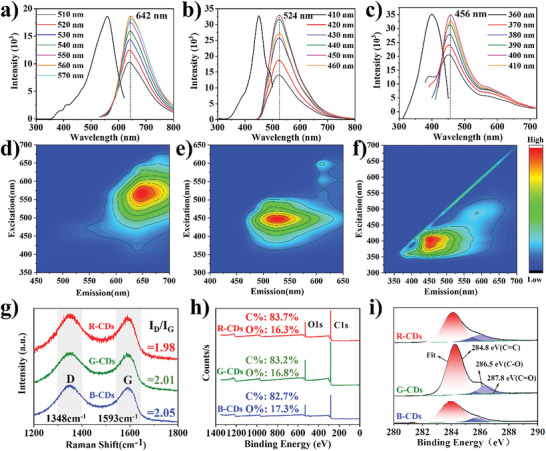
a–c) PL excitation and emission spectra and d–f) 3D FL spectra of RGB‐CDs in ethanol (*c* = 0.1 mg mL^−1^). g) Raman and h) XPS full survey spectra of RGB‐CDs. i) High‐resolution C1s spectra of RGB‐CDs.

The optical properties of the CDs were explored by ultraviolet–visible (UV−vis) and photoluminescence (PL) spectroscopy. The RGB‐CDs exhibited very similar absorption spectra because they were prepared from the same precursor (Figure S4, Supporting Information). In the UV region, significant absorption peaks were observed at 254 and 317 nm for the three CDs, which mainly reflected the *π*–π* transition in the aromatic carbon and the n–π* transition between the sp^2^ domains.^[^ ^27^, ^28^, ^29^, ^30^, ^31^, ^32^, ^33^, ^34^, ^35^ ^]^ Moreover, typical B absorption bands (278 nm) were observed for the B‐CDs, indicating the retention of the isolated aromatic structure within the carbon cores. In the lower‐energy region, unlike many other reported CDs, these three CDs samples did not give rise to absorptions from surface defect states, indicating that the surface defect state was not the main source of luminescence by these CDs.^[^ ^36^ ^]^ **Figure** **2**a–c shows fluorescence emission (PL) spectra for the RGB‐CDs, in which the emission maxima of the R‐CDs, G‐CDs, and B‐CDs appeared at *λ*_max_ = 642, 524, and 456 nm, respectively. The emission of these CDs was almost independent of the excitation wavelength. The calculated fluorescence quantum yields (QYs) were 38.0%, 36.4%, and 24.0% for RGB‐CDs, respectively. The 3D spectra of these CDs showed that there was only one best excitation light source, consistent with the fluorescence spectrum analysis, and there was a single emission center (Figure 2d–f).^[^ ^37^ ^]^

As shown in Figure S5 (Supporting Information), the RGB‐CDs gave rise to similar Fourier transform infrared (FT‐IR) spectra, indicating that they have similar chemical compositions and group distribution characteristics. The changes in the *I*_D_/*I*_G_ ratios and C=C bond contents determined by Raman and X‐ray photoelectron spectroscopy show that the sp^2^ carbon structure gradually decreased from R‐CDs to G‐CDs and then to B‐CDs, but there was little difference among the three CDs (Figure 2g–i; Figure S6 and Table S1, Supporting Information).^[^ ^27^ ^]^

**Figure 3:**
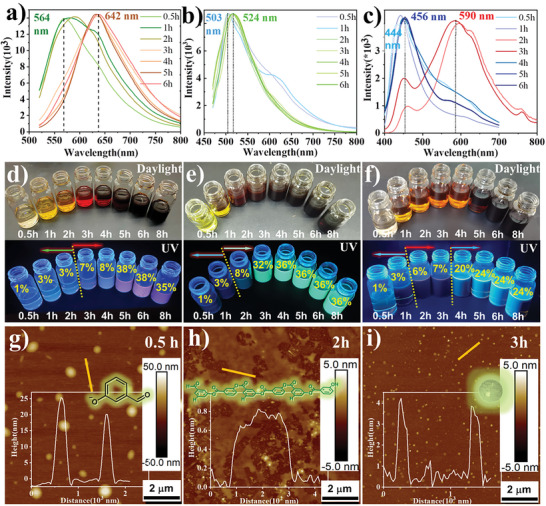
PL emission spectra of: a) R‐CDs, b) G‐CDs, and c) B‐CDs at different reaction times. Photos of the as‐prepared under different reaction times: d) R‐CDs, e) G‐CDs, and f) B‐CDs in daylight (upper) and UV light (bottom) and the change in QYs. g–i) AFM images of G‐CDs at different reaction times.

Three popular PL mechanisms have been proposed for CDs. One is based on band gap transitions in conjugated *π*‐domains (molecular state), and the other two are related to surface defects and the quantum size of CDs.^[^ ^7^, ^8^, ^9^, ^10^, ^11^, ^12^, ^13^, ^14^, ^15^, ^16^, ^17^, ^18^, ^19^ ^]^ In our case, we believe that molecular‐state fluorescence primarily controls photoluminescence because our samples do not show absorption of surface defect states, and the quantum size does not affect the fluorescence characteristics. To further explore CD formation and the relevant band gap tuning mechanism, we carried out time gradient experiments on the synthesis of three kinds of CDs and observed the changes in PL and QYs with synthesis time (**Figure** **3**).^[^ ^38^ ^]^ We found that the three CDs emitted distinct fluorescence even in the early stage of the reaction, and their maximum emission wavelengths were 564, 503, and 414 nm (Figure 3a–f). As the reaction progressed, the maximum fluorescence wavelength of the R,G‐CDs was slowly redshifted by 100 and 40 nm to 642 and 524 nm, respectively. The above changes in the fluorescence spectra suggest that the conjugated structure of fluorescent molecules gradually became larger in R,G‐CDs. In contrast with the R,G‐CDs, the B‐CDs showed two fluorescence centers, one at 456 nm and the other at 596 nm. The solution showed red fluorescence at 2 and 3 h of reaction. As the reaction progressed, the fluorescence at 596 nm gradually decreased, while the fluorescence intensity at 456 nm gradually increased. Finally, only the fluorescence at 456 nm remained, and the solution also changed from red to blue fluorescence. This change in fluorescence shows that the structure of the fluorophores changed again with the reaction.

Interestingly, during the formation of the RGB‐CDs, the QYs increased rapidly at a certain stage. For example, for G‐CDs, at a reaction time of 2 h, the maximum fluorescence wavelength was no different from that of the final G‐CDs, but the QY was only 8.0%. When the reaction time reached 3 h, the QY increased to 32%, and the maximum fluorescence wavelength did not change. Their fluorescence lifetime changed suddenly during the reaction process and remained consistent with the change in QY (Figure S7 and Table S2, Supporting Information). The R‐CDs and B‐CDs also showed similar fluorescence phenomena. Such optical experimental results indicate that fluorophores of some structure were generated first, and then these fluorophores gathered together to inhibit the energy loss caused by molecular vibration, thus improving the fluorescence efficiency. This hypothesis was also confirmed by the results of time‐dependent atomic force microscopy (AFM) (Figure 3g–i and Figure S8, Supporting Information). At the beginning of the reaction, a large number of spherical structures with heights of 30–50 nm were observed, which may have formed by the aggregation of *m*‐hydroxybenzaldehyde, but fluorescence was hardly observed. After the reaction progressed, these larger spherical structures were replaced by amorphous structures with a height of less than 1 nm, indicating the consumption of *m*‐hydroxybenzaldehyde caused by chemical or carbonization reactions. At this time, the fluorescence efficiency was still very low, but the wavelength of fluorescence had reached the maximum. When the reaction reached the late stage, a large number of spherical structures with a height of 3–4 nm were observed, and the fluorescence efficiency was also greatly improved, indicating that the formation of CDs can improve the quantum yield of the fluorophore.

**Figure 4:**
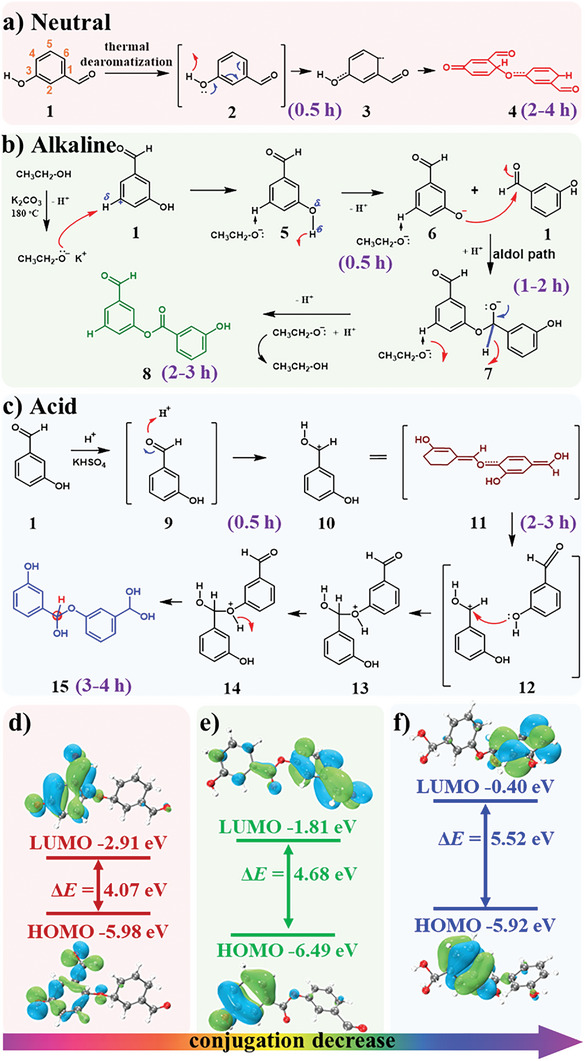
Schematic illustration of the synthesis procedures of: a) R‐CDs, b) G‐CDs, and c) B‐CDs under different reaction conditions. d–f) The change in energy gap between the HOMO and LUMO of the unit structure of the fluorophores (1, 8, and 15) of the R‐CDs, G‐CDs, and B‐CDs.

To investigate molecular transformations during the production of three colored CDs and determine the reaction mechanism, dynamic nuclear magnetic resonance spectroscopy (NMR) experiments were conducted at different reaction times, including 0.5 h, 1–6 h (tests every 1 h), and details of these NMR data are presented in Figures S9–S11 (Supporting Information). As depicted in **Figure** **4**a, the reactant of *m*‐hydroxybenzaldehyde 1 exhibited good thermal de‐aromatization characteristics for the synthesis of quinone‐like isomer 3, which produced a regular *π*–*π* conjugation of structure unit 4 (Figures S12 and S13, Supporting Information). This structural unit change can be observed in the PL spectra in Figure 3a for the R‐CDs, with the maximum emission peak changing from 564 to 642 nm. For the K_2_CO_3_‐induced synthesis of green CDs, as shown in Figure 4b, the alcohol solvent was primarily ionized into potassium ethoxide salt, and this base smoothly stabilized the 5‐site of *m*‐hydroxybenzaldehyde 1 and deprotonated the phenol into 6, leading to an aldol path to semiacetal intermediate 7, which was finally esterified into the final green CDs unit 8. The aldol reaction process was proven by the PL spectra of Figure 3c because the maximum emission peak changed from 503 to 524 nm over a reaction time of 2 h. The five‐site esterification was also confirmed by the four‐site and six‐site carbon data in the ^13^C‐NMR spectrum (*δ* = 119.88 and 122.81 ppm, details are shown in Figures S14 and S15, Supporting Information). For the KHSO_4_‐induced synthesis of blue CDs, as shown in Figure 4c, the aldehyde group was acidized first under the reaction conditions, leading to the tertiary carbon cation center of 10 (this reaction step occurred along with the PL spectral change from 456 to 596 nm in Figure 3e). Then, the carbon cation abstracted phenol and formed the stable semiacetal product 15 under acidic conditions, leading to the PL peak shifting back from 596 nm in Figure 3f. The ^1^H NMR (*δ* = 5.28 ppm) and ^13^C NMR (*δ* = 103.08 ppm) spectra also confirmed the semiacetal structural unit 15 in these blue CDs in Figures S16 and S17 (Supporting Information).

**Figure 5:**
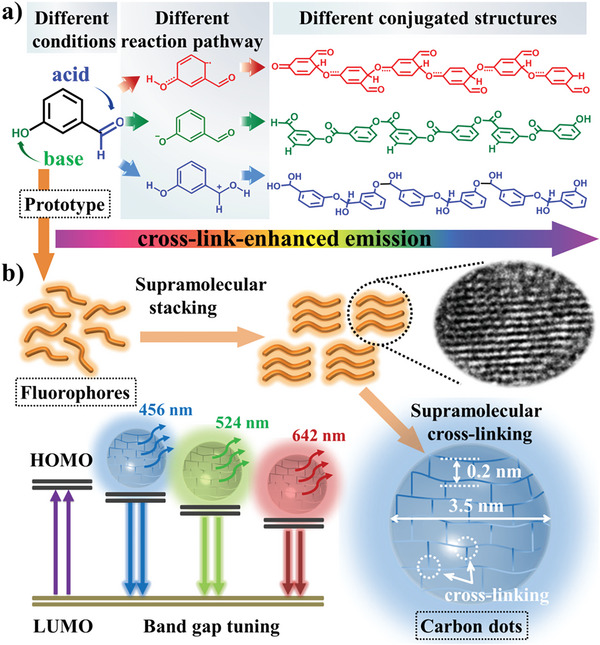
a) Mechanisms of CDs formation under different reaction conditions. b) The fluorescence mechanism diagram for CDs with tunable PL emission.

Based on the above results, the unit structure of the fluorophores might be dyads 1, 8, and 15 for RGB‐CDs. By density functional theory (DFT) simulations of these unit structures,^[^ ^39^, ^40^ ^]^ it was proven that their energy gaps (*E*g) were 4.07, 4.68, and 5.52 eV (Figure 4d), respectively, and the conjugated structures of the unit decreased, which led to a blue shift in the fluorescence and the generation of red, green, and blue emission under UV light. In general, the base acted on the hydroxyl group, and the acid acted as the aldehyde group during the reaction, leading the precursor to take different reaction paths and form fluorescent groups with different conjugated structures. Moreover, the molecular length along the long axis of these dyads was ≈1.2 nm. The average particle size of these CDs determined by TEM was 3.5 nm, indicating that the fluorophores were condensed by up to six *m*‐hydroxybenzaldehyde units (**Figure** **5**a). Although the fluorescence QYs of these oligomeric fluorophores was very low (≈7–8%), their fluorescence QYs were greatly improved (≈20–30%) after supramolecular aggregation to form CDs with a layer spacing of 0.2 nm. Supramolecular aggregation could be used to enhance the photoluminescence by inhibiting the motion of the fluorescence center, similar to the supramolecular cross‐link‐enhanced emission effect as the SCEE effect.^[^ ^41^, ^42^, ^43^ ^]^

The RGB‐CDs have unique optical properties and excellent color stability (Figure S18, Supporting Information). They have the potential to be used as fluorescent agents in the field of light‐emitting devices (LEDs). In addition, ethanol solutions of R‐CDs, G‐CDs, and B‐CDs were dropped on an InGaN‐based LED chip and dried to obtain red, green, and blue LEDs (Figure S19, Supporting Information). The CIE coordinates of R‐LED, G‐LED, and B‐LED are (0.46, 0.42), (0.36, 0.52), and (0.18, 0.21), respectively. Furthermore, R‐CDs, G‐CDs, and B‐CDs were mixed on the LED chip in a ratio of 1:1:2 to produce a white LED (W‐LED). The PL emission spectrum of this W‐LED is panchromatic emission at 400–700 nm, and the CIE coordinates of W‐LED were (0.35, 0.36).

## 3 Conclusion

In summary, we synthesized multicolor emission CDs with *m*‐hydroxybenzaldehyde as the precursor and analyzed the mechanisms of band gap tuning and formation of CDs through a time‐dependence experiment in the CDs synthesis process. Dynamic NMR experiments indicated that different reaction conditions led to different reaction paths and finally produced molecular fluorophores with different conjugated structures. The dynamic spectrum and AFM analysis showed that although fluorophores that could emit light independently were formed at the initial stage of the reaction, their efficiency was very low. When CDs were formed through cross‐linking, their fluorescence efficiency was greatly improved. At present, we cannot determine whether this cross‐linking occurs through covalent bonds or noncovalent bonds, so further studies are required. Our findings can potentially help understand the PL mechanism of CDs and inspire a novel synthetic design to obtain multicolor emission with tailored properties.

## Conflict of Interest

The authors declare no conflict of interest.

## Supporting information

Supporting Information

## Data Availability

The data that support the findings of this study are available in the supplementary material of this article.
