# Supplementary material for: Formation and Band Gap Tuning Mechanism of Multicolor Emissive Carbon Dots from m‐Hydroxybenzaldehyde
Source: Adv Sci (Weinh). 2023 Apr 21;10(18):2300543. doi: 10.1002/advs.202300543 (PMC10288221; doi:10.1002/advs.202300543)
Supplement: Supplementary file 1 — Supporting Information [file ADVS-10-2300543-s001.pdf]

ADVANCED SCIENCE
Open Access

Supporting Information

for *Adv. Sci.*, DOI 10.1002/advs.202300543

Formation and Band Gap Tuning Mechanism of Multicolor Emissive Carbon Dots from *m*-Hydroxybenzaldehyde

Yan Li, Can Liu, Hao Sun, Menglin Chen, Defa Hou, Yunwu Zheng, Haijiao Xie, Bei Zhou* and Xu Lin**

Supporting Information

Formation and Band Gap Tuning Mechanism of Multicolor emissive Carbon Dots from *m*-Hydroxybenzaldehyde

Yan Li[a], *Can Liu*[a]*, *Hao Sun*[a], *Menglin Chen*[a], *Defa Hou*[a], *Yunwu Zheng*[a], *Xie Haijiao*[b], *Bei Zhou*[a]* *and Xu Lin*[a]*

[a] National Joint Engineering Research Center for Highly-Efficient Utilization Technology of Forestry Resources; Southwest Forestry University, 300 Bailong Road, Kunming 650224, Yunnan Province, China;
[b] Hangzhou Yanqu Information Technology Co., Ltd., Y2, 2nd Floor, Building 2, Xixi Legu Creative Pioneering Park, No. 712 Wen'er West Road, Xihu District, Hangzhou 310003, Zhejiang Province, China;
E-mail: linxu@swfu.edu.cn

Materials

m-hydroxybenzaldehyde (99.0%), ethanol (99.7%), potassium hydrogen sulfate, potassium carbonate are provided by Shanghai Titan Science Co., Ltd. Unless otherwise stated, all reagents are used as is and no further purification is required.

Methods

Transmission electron microscopy (TEM) images was carried out using a FEI Tecani G2 F20 operating at an acceleration voltage of 200 kV. UV-vis spectra were recorded with a Shimadzu UV-2600 spectrometer. Fluorescence measurements were collected using a Shimadzu fluorescence spectrophotometer RF-6000. The Fourier transform infrared (FT-IR) spectra were obtained in transmission mode on a Thermal Scientific Nicolet iS5 spectrometer (Waltham, MA, USA) with the KBr pellet technique, and 8 scans at a resolution of 1 cm^{-1} were accumulated to obtain one spectrum. X-ray photoelectron spectroscopy (XPS) was investigated by using K-Alpha spectrometer with a mono X-Ray source Al Kα excitation (1486.6 eV). Binding energy calibration was based on C1s at 284.7 eV. Use HORIBA Scientific LabRAM HR Evolutio for Raman analysis. QYs of the obtained three CDs were determined by a relative method. Specially, quinine sulfate (QY = 55% in 0.1 M H2SO4) was selected as the reference for the blue emission, rhodamine 6G (QY = 95% in ethanol) for the green emission, and rhodamine B (QY = 56% in ethanol) for the red emission. Atomic force microscope (AFM) imaging was carried out using Bruker Dimension Icon. Fluorescence lifetime recorded by Edinburgh FLS1000. The ^{1}H-NMR and ^{13}C-NMR spectra were obtained from Bruker Avance NEO 400MHz in Germany.

Synthesis of R-CDs, G-CDs and B-CDs

m-Hydroxybenzaldehyde (2.0 g) was dissolved in 10 mL ethanol and then transferred to a polytetrafluoroethylene-lined autoclave. A red fluorescent suspension (pH=7) was obtained upon heating in a 180 °C oven for 8 hours and natural cooling to

room temperature. In addition, using *m*-hydroxybenzaldehyde (2.0 g) as the raw material, potassium carbonate and potassium bisulfate were added, and green (pH=9) and blue (pH=3) fluorescent suspensions were obtained by the same method. After the pH value of the suspensions is adjusted to 7, the crude products were purified by silica gel column chromatography, and the eluent was a mixture of dichloromethane and ethanol. The process was repeated three times to remove excess impurities and unreacted precursors, and finally, three purified products of R-CDs, G-CDs and B-CDs were obtained.

DFT calculation

The Gaussian 09 software calculated geometry optimizations in the ground state and energy levels of the molecular orbits. The B3L YP-D3/6-31G(d,p) level of theory was used to thoroughly optimize the structures of the substances under study. The optimized structures performed at the same level for their vibrational frequencies. By confirming that each vibrational frequency existed in the structures, the local energy minimum on the potential energy surface was identified. Calculations were done at the same level for the energy of the HOMOs and LUMOs as well as their gaps.

Supporting Figures

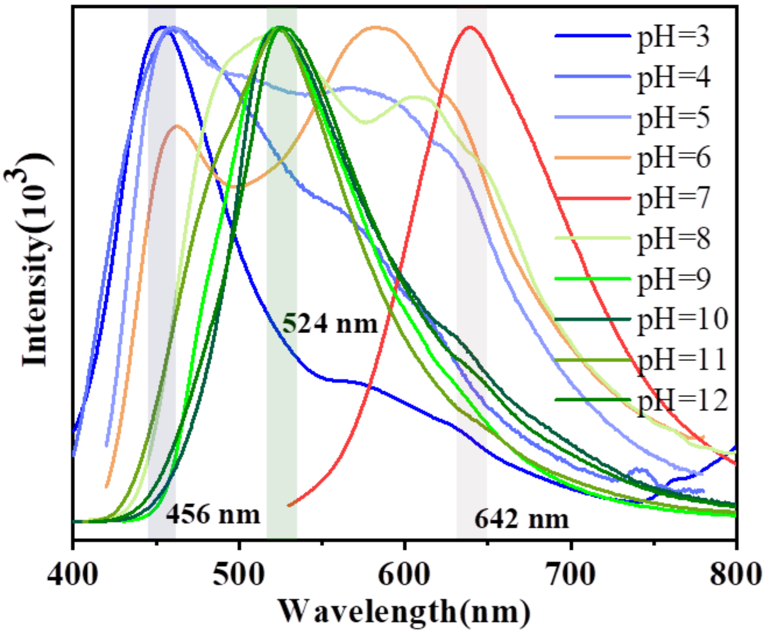

Figure. S1 PL emission spectra of multicolor CDs prepared at different pH values.

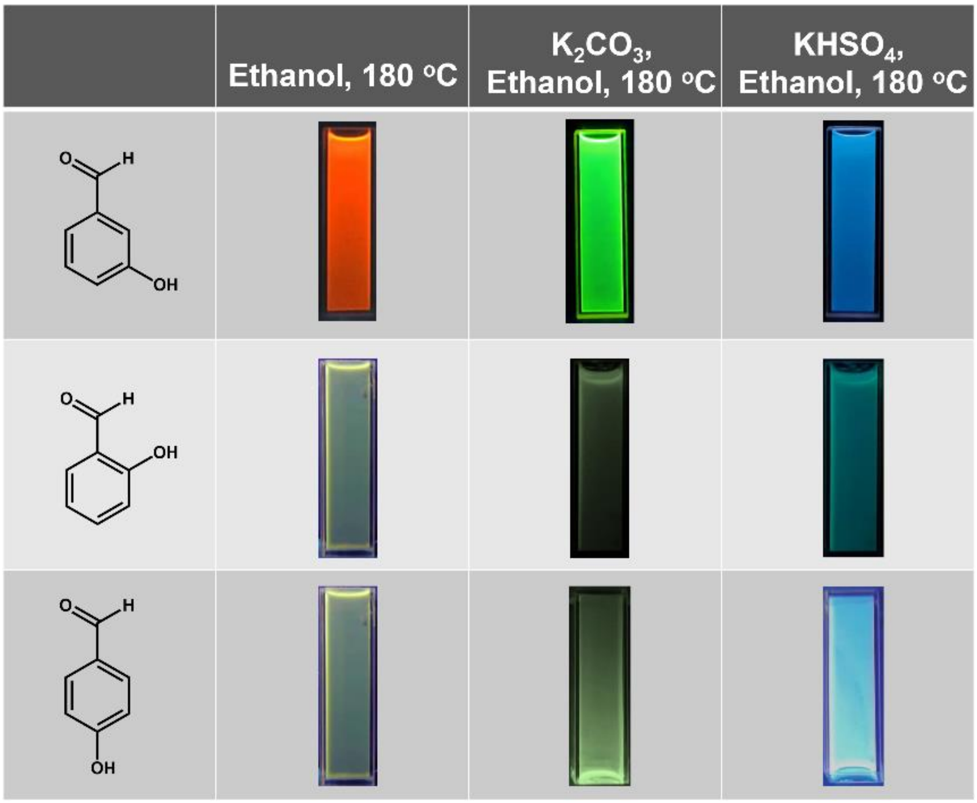

Figure. S2 The carbon dots were prepared by the reaction of hydroxybenzaldehyde with different structures under three conditions. Under the UV irradiation of λ_{ex} = 365 nm, the luminescence in ethanol was photographed.

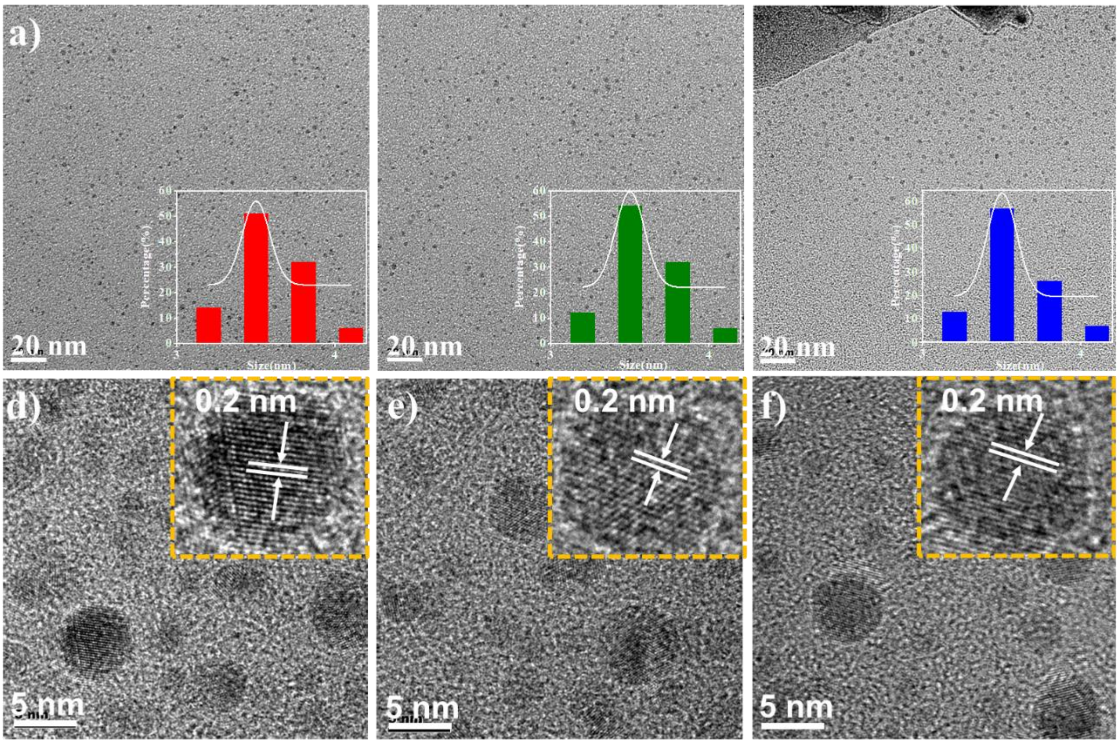

Figure. S3 TEM images of a) R-CDs, b) G-CDs, and c) B-CDs. The average particle sizes of R-CD, G-CD, and B-CD were 3.50 ± 0.19 nm, 3.50 ± 0.21 nm and 3.50 ± 0.25 nm, respectively. Insets: histograms and Gauss fittings of particle size distribution d-f) Enlarged TEM images of RGB-CDs.

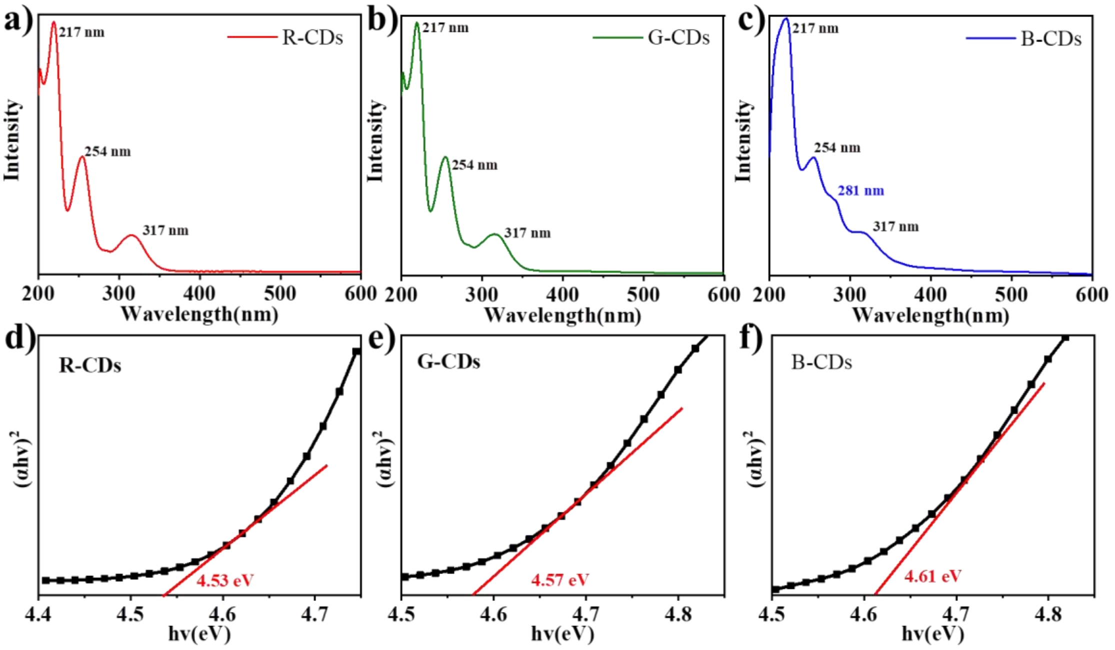

Figure. S4 UV/Vis absorption spectra of a) R-CDs, b) G-CDs, and c) B-CDs in ethanol solution (*c* = 0.1 mg/mL) d-f) plot of $(\alpha h\nu)^2$ versus photon energy for RGB-CDs.

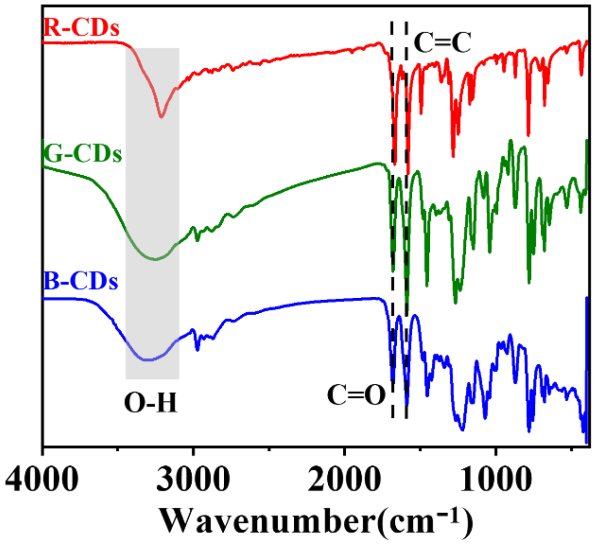

Figure. S5 FT-IR spectra of R-CDs, G-CDs, and B-CDs.

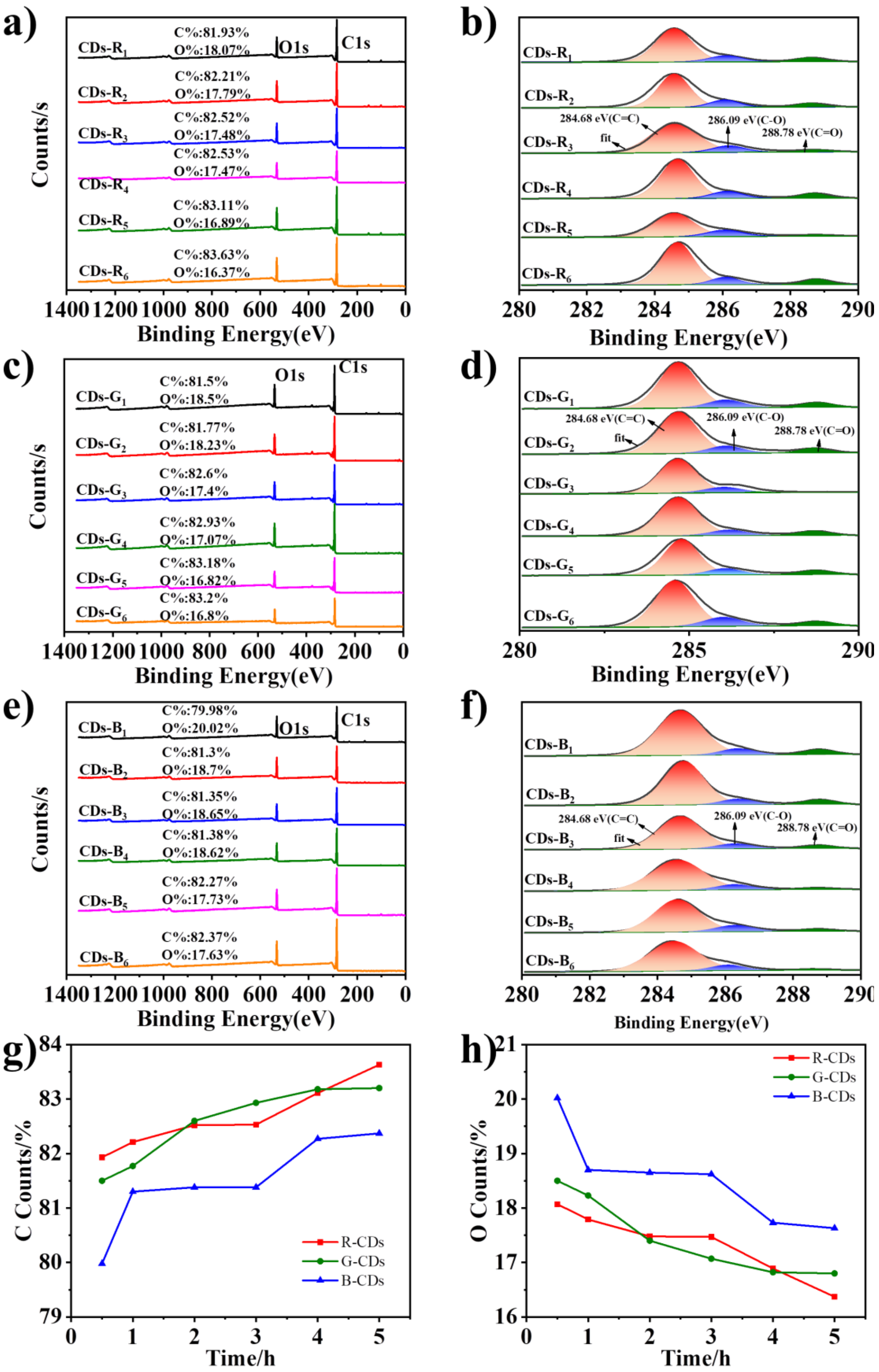

Figure. S6 a,c,e) XPS full survey spectra of and b,d,f) high-resolution C 1s spectra of RGB-CDs at different reaction time. g,h) Change of carbon and oxygen contents of RGB-CDs at different reaction time.

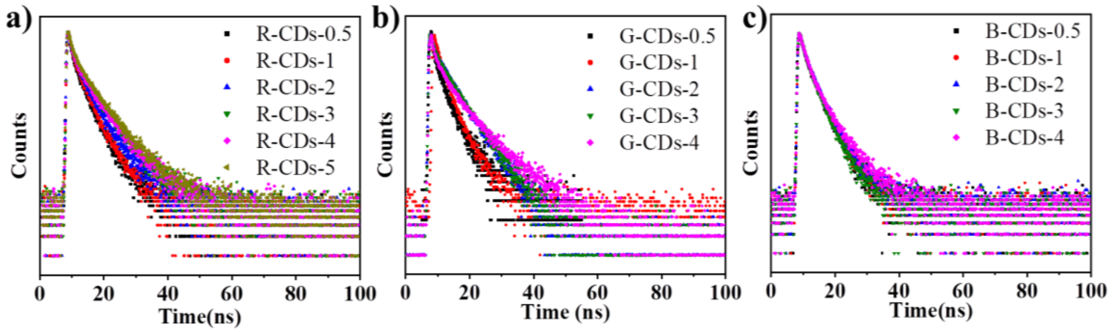

Figure. S7 Photoluminescent decays of a) R-CDs b) G-CDs c) B-CDs at different reaction time in ethanol solution (c = 0.1 mg/mL).

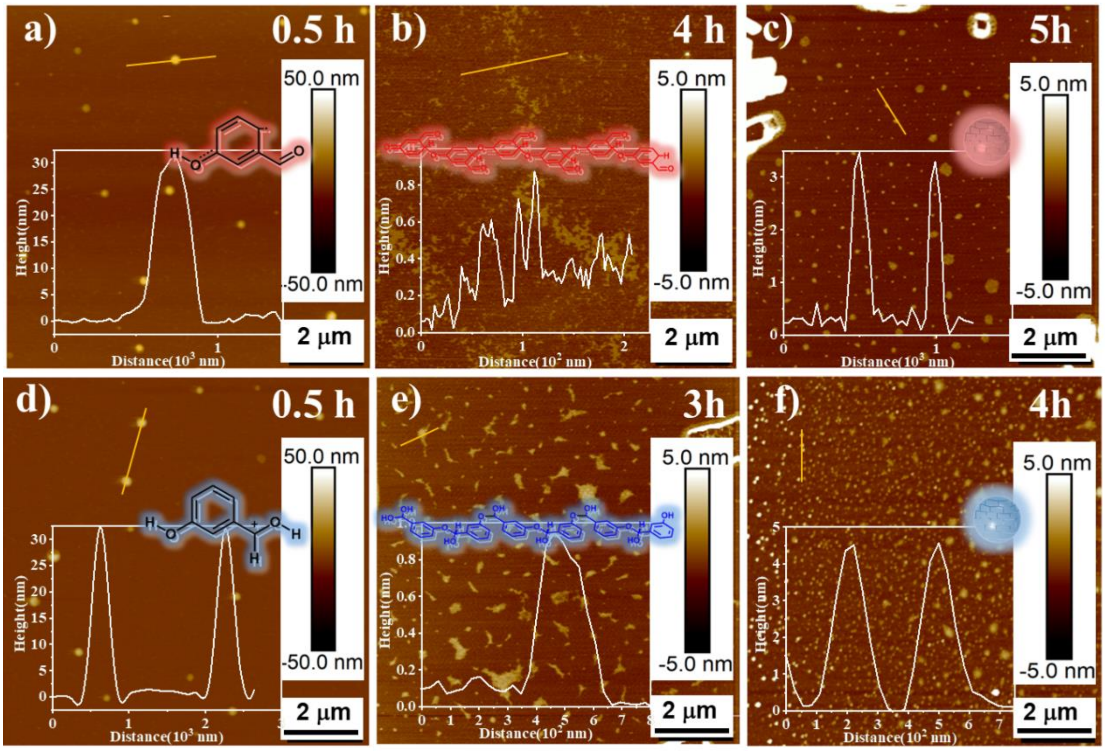

Figure. S8 AFM images of (a-c) R-CDs and (d-f)B-CDs at different reaction times spin-coated from ethanol solution (c = 1.0 mg/mL).

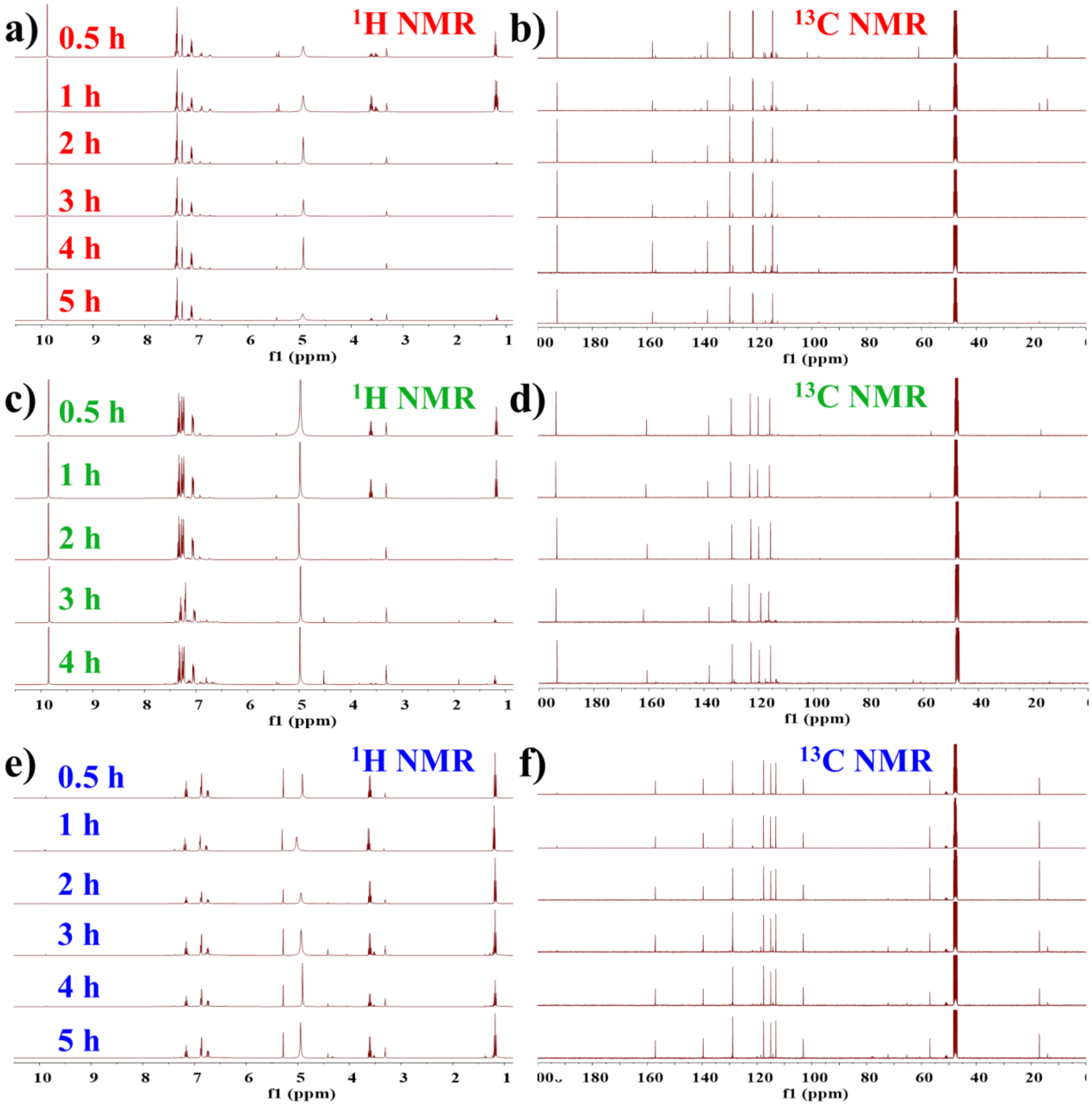

Figure. S9 ^{1}H-NMR and ^{13}C-NMR spectra of a-b) R-CDs, c-d) G-CDs, and e-f) B-CDs at different reaction times in MeOD.

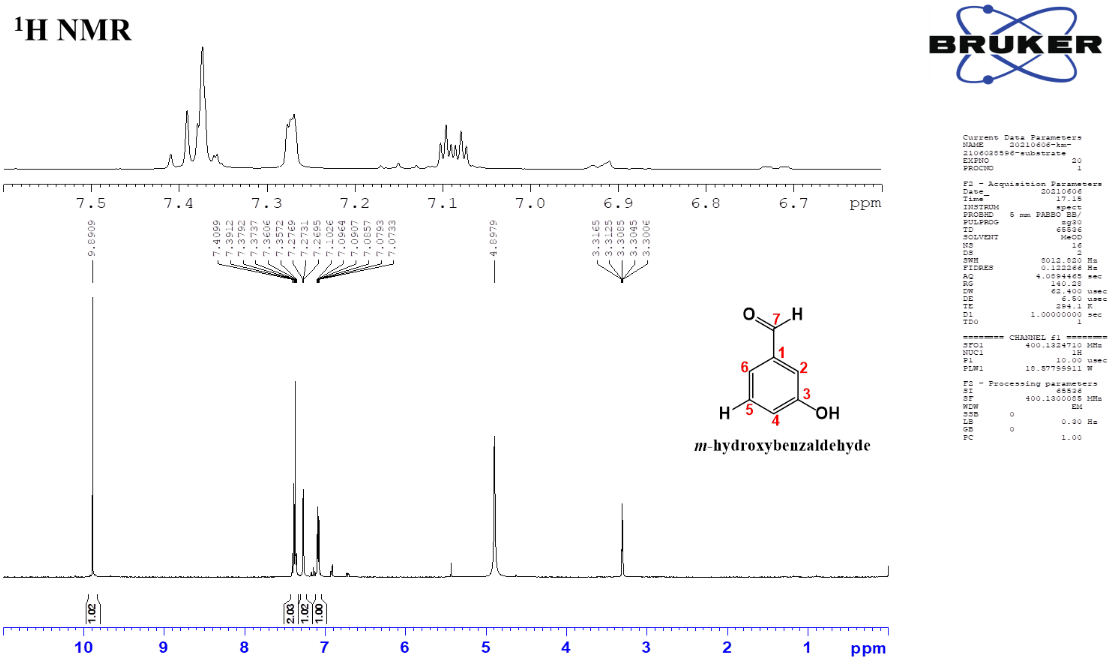

Figure. S10 ^{1}H-NMR spectra of *m*-hydroxybenzaldehyde in MeOD.

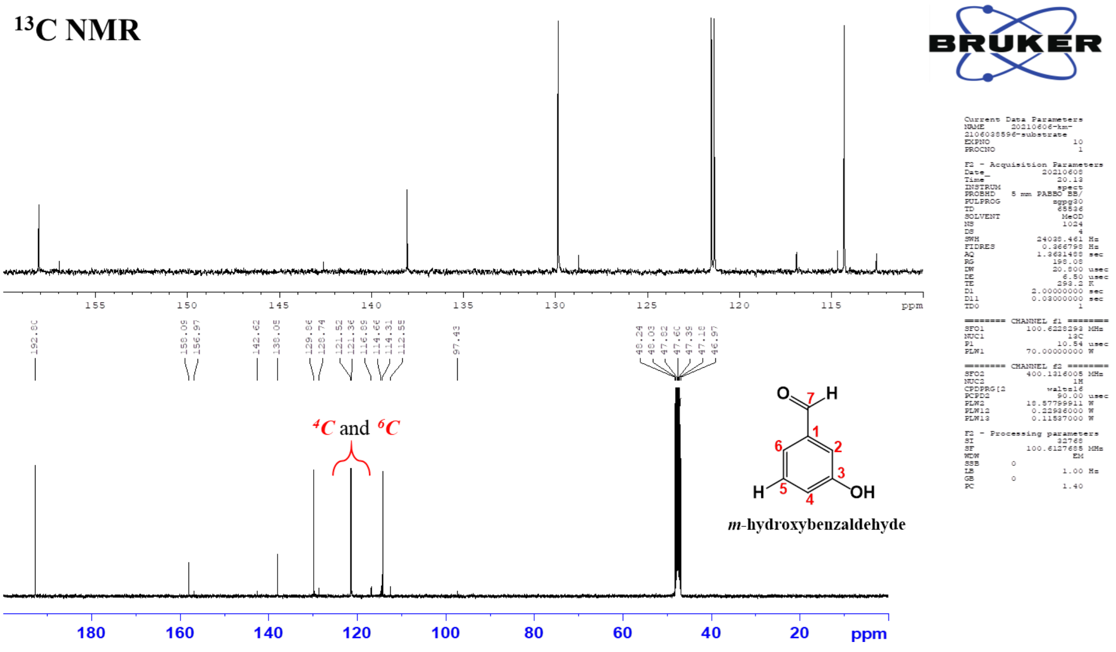

Figure. S11 ^{13}C-NMR spectra of *m*-hydroxybenzaldehyde in MeOD.

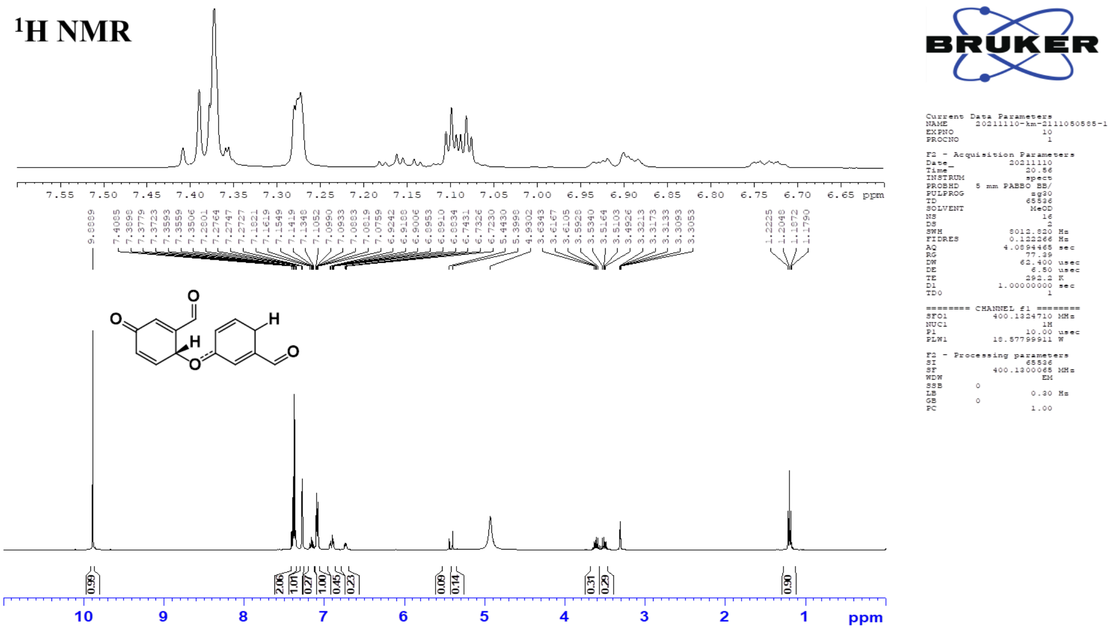

Figure. S12 ^{1}H-NMR spectra of R-CDs (neutral reaction condition) in MeOD.

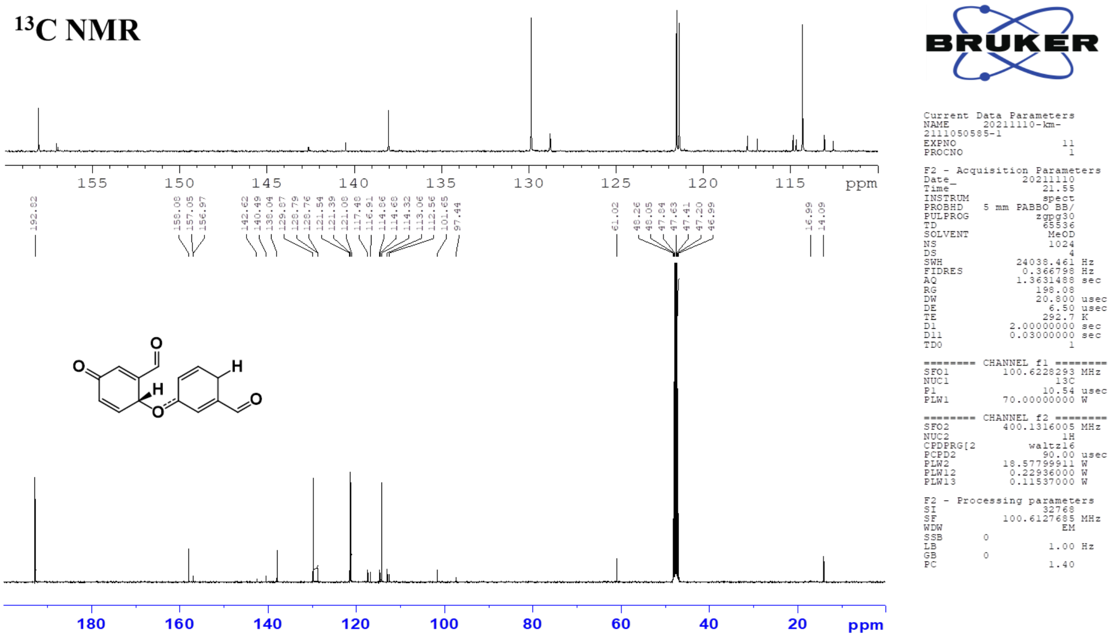

Figure. S13 ^{13}C-NMR spectra of R-CDs (neutral reaction condition) in MeOD.

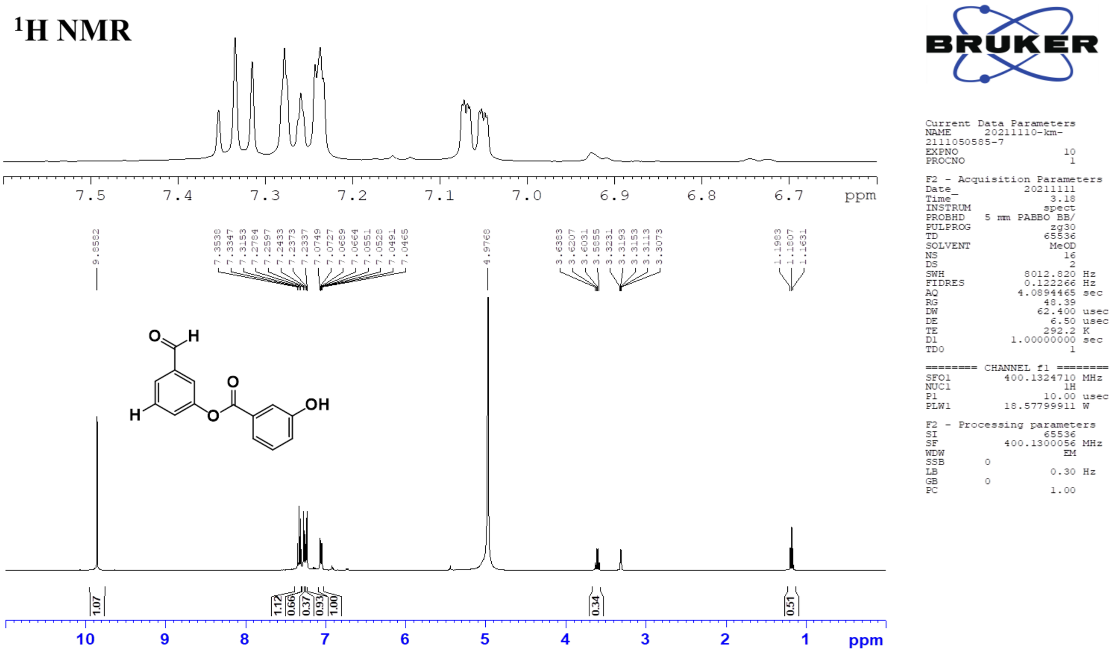

Figure. S14 ^{1}H-NMR spectra of G-CDs (alkaline reaction condition) in MeOD.

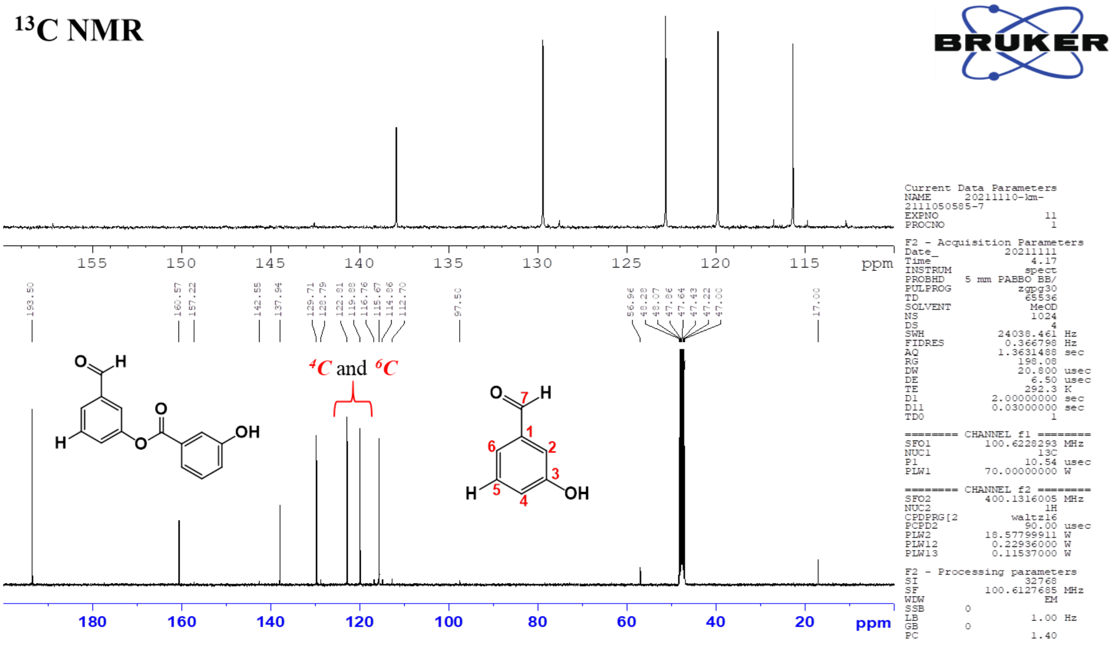

Figure. S15 ^{13}C-NMR spectra of G-CDs (alkaline reaction condition) in MeOD.

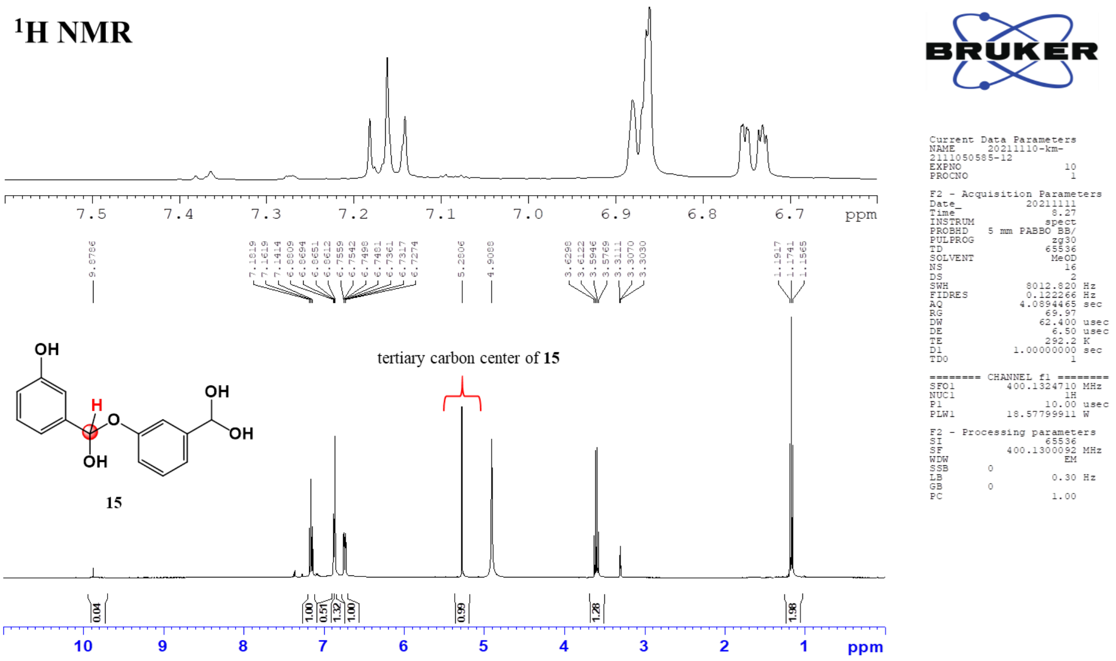

Figure. S16 ^{1}H-NMR spectra of B-CDs (acid reaction condition), in MeOD.

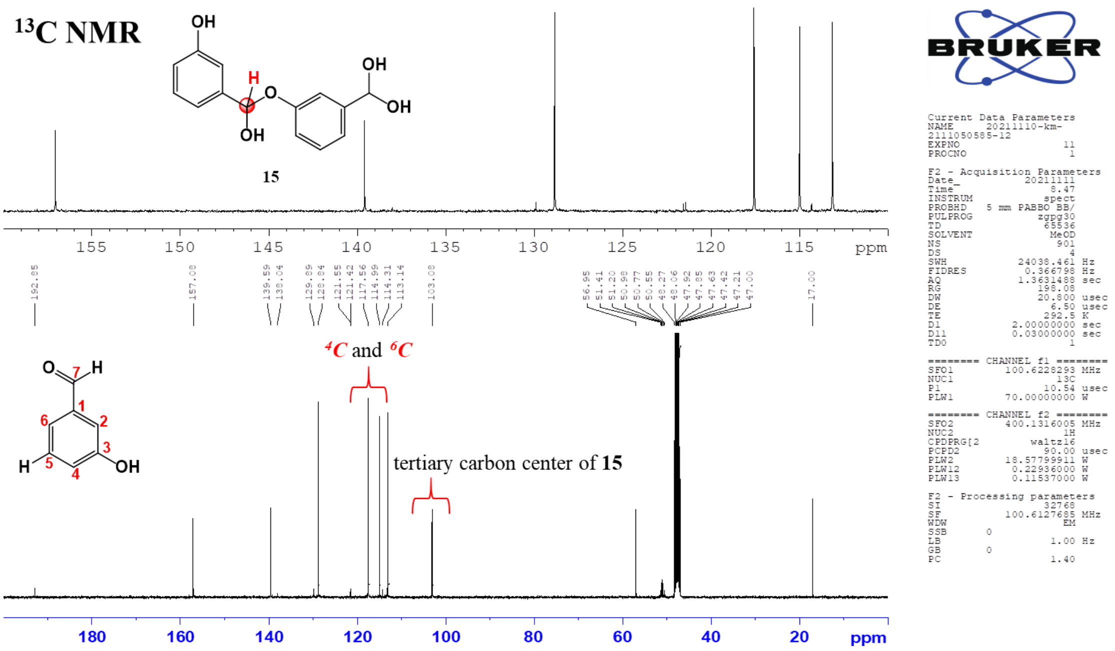

Figure. S17 ^{13}C-NMR spectra of B-CDs (acid reaction condition) in MeOD.

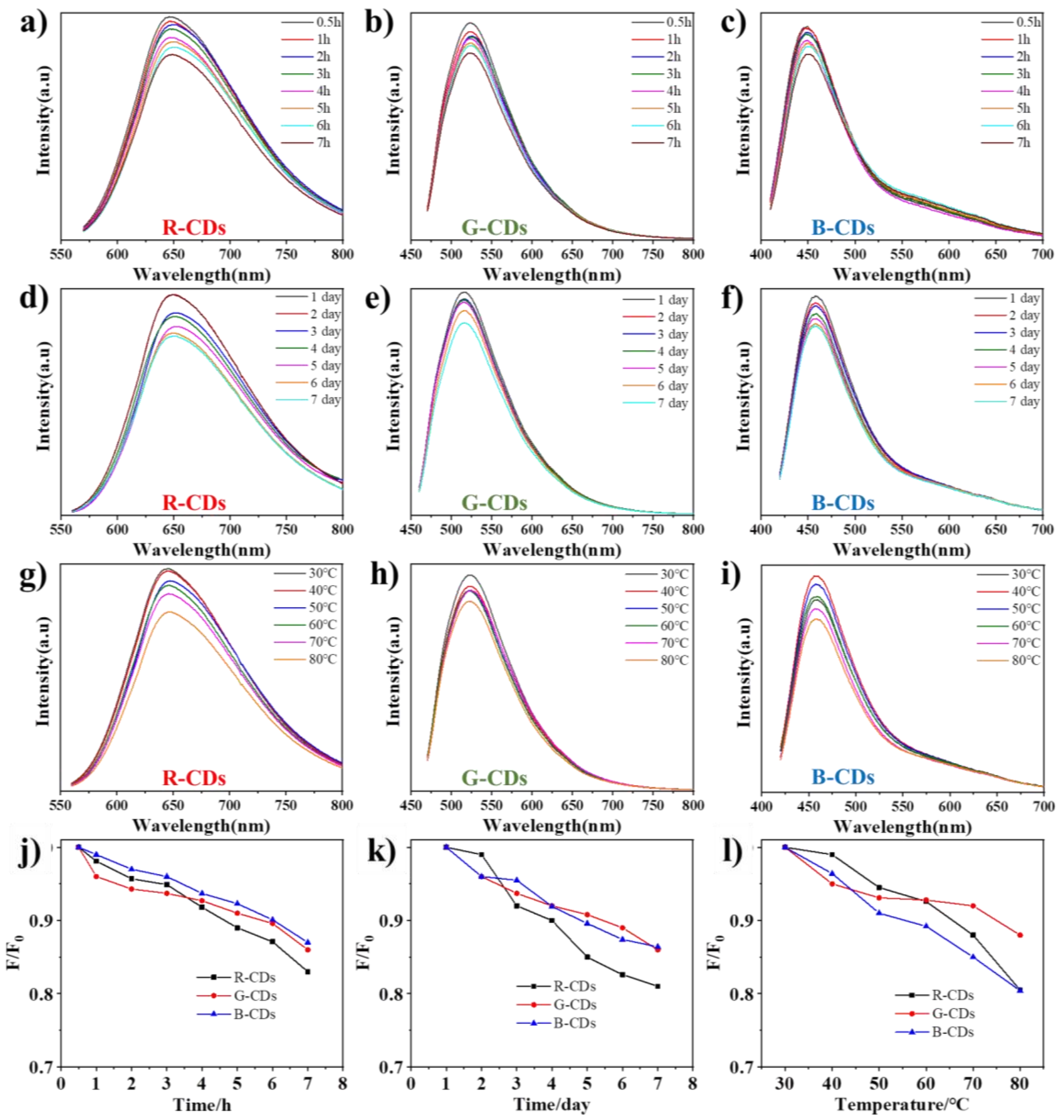

Figure. S18 PL emission spectra of RGB-CDs at different durations under visible light (a-c) and at different durations under ultraviolet light (d-f) in ethanol solution. (h-j) PL emission spectra of RGB-CDs in a water bath at different temperatures in ethanol solution. Decay curve of FL intensity of RGB-CDs with increasing time (j) UV irradiation (λex = 365 nm) and (k) visible, (l) Decay curve of FL intensity of three CDs with kincreasing temperature (initial temperature 30°C).

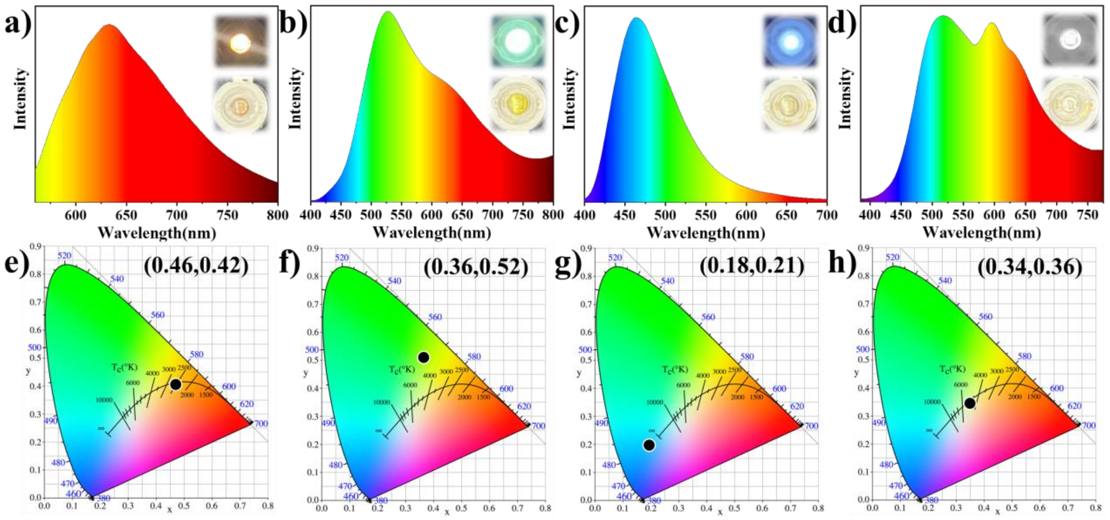

Figure. 19 The EL emission spectrum of the a) R-LED, b) G-LED, c) B-LED and d) W- LED. The insets show optical images of the LEDs in the "on" (top) and "off" (bottom) states. The CIE containing the color coordinates of the e) R-LED, f) G-LED, g) B-LED and h) W-LED.

Table S1. Elemental proportions and chemical bonds in RGB-CDs at different reaction times.

**R-CDs**	**0.5 h**	**1.0 h**	**2.0 h**	**3.0 h**	**4.0 h**	**5.0 h**
C1s (%)	81.93	82.21	82.52	82.53	83.11	83.63
O1s (%)	18.07	17.79	17.48	17.47	16.89	16.37
C=C (%)	86.78	87.13	87.63	88.76	89.47	89.70
C-O (%)	10.95	10.35	10.82	7.54	8.44	7.73
C=O (%)	2.27	2.52	1.55	3.70	2.09	2.57
**G-CDs**	**0.5 h**	**1.0 h**	**2.0 h**	**3.0 h**	**4.0 h**	**5.0 h**
C1s (%)	81.50	81.77	82.6	82.93	83.18	83.20
O1s (%)	18.50	18.23	17.40	17.07	16.82	16.80
C=C (%)	85.30	85.91	85.93	86.83	87.10	87.60
C-O (%)	10.45	10.79	12.38	9.22	8.70	8.90
C=O (%)	4.25	3.30	1.69	3.95	4.20	3.50
**B-CDs**	**0.5 h**	**1.0 h**	**2.0 h**	**3.0 h**	**4.0 h**	**5.0 h**
C1s (%)	79.98	81.30	81.35	81.38	82.27	82.37
O1s (%)	20.02	18.70	18.65	18.62	17.73	17.63
C=C (%)	84.83	85.13	85.58	86.09	86.34	86.87
C-O (%)	10.32	9.89	10.21	12.86	9.97	12.86
C=O (%)	4.85	4.98	4.21	1.05	3.69	0.27

Table S2. Photoluminescent decays of RGB-CDs at different reaction time

	**0.5 h**	**1.0 h**	**2.0 h**	**3.0 h**	**4.0 h**	**5.0 h**
**R-CDs** (ns)	3.370	3.590	4.260	5.140	5.160	5.780
**G-CDs** (ns)	3.109	4.459	4.858	5.352	5.478	-
**B-CDs** (ns)	3.390	3.486	3.666	3.762	4.042	-
